# Salivary Protein Panel to Diagnose Systolic Heart Failure

**DOI:** 10.3390/biom9120766

**Published:** 2019-11-22

**Authors:** Xi Zhang, Daniel Broszczak, Karam Kostner, Kristyan B Guppy-Coles, John J Atherton, Chamindie Punyadeera

**Affiliations:** 1 Saliva and Liquid Biopsy Translational Research Team, School of Biomedical Sciences, Institute of Health and Biomedical Innovation, Queensland University of Technology, Brisbane, Queensland 4059, Australia; x81.zhang@qut.edu.au; 2 School of Biomedical Sciences, Institute of Health and Biomedical Innovation, Queensland University of Technology, Brisbane, Queensland 4059, Australia; daniel.broszczak@qut.edu.au; 3 Department of Cardiology, Mater Adult Hospital, Brisbane, Queensland 4101, Australia; k.kostner@uq.edu.au; 4 Cardiology Department, Royal Brisbane and Women’s Hospital and University of Queensland School of Medicine, Brisbane, Queensland 4029, Australia; Kristyan.Guppy-Coles@health.qld.gov.au (K.B.G.-C.); John.Atherton@health.qld.gov.au (J.J.A.)

**Keywords:** systolic heart failure, cardiovascular diseases, saliva, biomarker, diagnosis, screen, prognosis

## Abstract

Screening for systolic heart failure (SHF) has been problematic. Heart failure management guidelines suggest screening for structural heart disease and SHF prevention strategies should be a top priority. We developed a multi-protein biomarker panel using saliva as a diagnostic medium to discriminate SHF patients and healthy controls. We collected saliva samples from healthy controls (n = 88) and from SHF patients (n = 100). We developed enzyme linked immunosorbent assays to quantify three specific proteins/peptide (Kallikrein-1, Protein S100-A7, and Cathelicidin antimicrobial peptide) in saliva samples. The analytical and clinical performances and predictive value of the proteins were evaluated. The analytical performances of the immunoassays were all within acceptable analytical ranges. The multi-protein panel was able to significantly (*p* < 0.001) discriminate saliva samples collected from patients with SHF from controls. The multi-protein panel demonstrated good performance with an overall diagnostic accuracy of 81.6% (sensitivity of 79.2% and specificity of 85.7%) when distinguishing SHF patients from healthy individuals. In conclusion, we have developed immunoassays to measure the salivary concentrations of three proteins combined as a panel to accurately distinguish SHF patients from healthy controls. While this requires confirmation in larger cohorts, our findings suggest that this three-protein panel has the potential to be used as a biomarker for early detection of SHF.

## 1. Introduction

Systolic heart failure (SHF) is a severe life-threatening clinical syndrome with symptoms that can occur both at rest and on exertion. SHF occurs when a person’s heart is unable to pump sufficient blood to the body due to an abnormality of cardiac structure or function, causing symptoms such as dyspnea, fatigue, dizziness, and edema [1]. Heart failure affects approximately 38 million individuals world-wide [2]. Due to an ageing and growing population, heart failure prevalence is increasing globally [3]. SHF is caused by damage to cardiomyocytes leading to the inability of the heart to pump sufficient blood to the body [4]. Heart failure is the leading cause of hospitalization in the elderly, with mortality rates higher than most solid-organ cancers [5]. The lack of available screening methods for SHF hinders early detection, despite the availability of inexpensive treatments that reduce the risk of developing SHF and improving long-term survival outcomes [6].

To address this problem, recent focus has been on developing novel diagnostic methods to screen for SHF. Previous studies have demonstrated that the measurement of B-type natriuretic peptide (BNP) in blood is highly accurate for excluding SHF [7,8,9]. Other proteins, such as galectin-3 and cardiac troponin I, also have the potential to be used in routine clinical practice to manage patients with SHF [10,11]. A limitation of the above-mentioned protein assays is that blood is required as the diagnostic medium, requiring individuals to present at a medical facility and have blood collected by a trained phlebotomist, a more challenging prospect in rural and resource limited settings.

Saliva as an alternative diagnostic medium is gaining attention due to its ease of collection, non-invasive nature, and the ability of multiple sample collections per patient at a single time point. Moreover, saliva collection reduces risk for contracting infections and contains about 30% of the biomolecules that are present in blood [12,13,14,15,16,17,18]. We were able to detect the SHF biomarkers N-terminal pro b-type natriuretic peptide (NT-proBNP) [15] and galectin-3 [18] in human saliva samples in previous studies. In addition, we have previously detected high levels of Kallikrein-1 (KLK1), protein S100-A7 (S100A7), and Cathelicidin antimicrobial peptide (CAMP) in saliva samples from SHF patients compared to controls using sequential windowed acquisition of all theoretical fragment ion mass spectra (SWATH-MS) [16]. However, the relatively higher costs and the sample preparation/measurement time for SWATH-MS limits the use of mass spectrometry as a screening tool in large population-based screening studies [19,20]. An efficient, effective, and highly accurate detection method that requires minimal sample processing/preparation time is preferred. Immunoassays, particularly enzyme-linked immunosorbent assays (ELISA), have all the above-mentioned properties where antibodies for targets of interest are available. ELISAs can also be easily integrated into the existing infrastructure in hospital clinical laboratories. As a result, once these biomarkers are extensively validated in a multi-clinical setting, these can then easily be implemented within a clinical workflow.

The aims of this study were to: Develop a robust immunoassay to detect S100A7, CAMP, and KLK-1 in saliva samples; and determine the sensitivity and specificity of the three proteins combined as a panel to diagnose SHF.

## 2. Materials and Methods

### 2.1. Participants and Saliva Sample Collections

The study complies with the 2013 Declaration of Helsinki [21] and Australian Code for Responsible Conduct of Research [22]. Research ethics approval for saliva sample collection was obtained prior to collection. We obtained ethics approvals from the human research ethics committee of Mater Adult Hospital (approval number: HREC/13/MHS/142 (1806QA)), The University of Queensland (approval number: 2009000779) and Queensland University of Technology (approval number: 1400000616) for this study. All study participants were >18 years of age and provided informed written consent prior to inclusion of this study. To distinguish a receiver operating curve (ROC) with an area under the curve (AUC) of 0.85 from an ROC curve of no diagnostic value (AUC of 0.5) with 80% power and significance of 0.05, a minimum sample size of 100 patients with SHF is required to prove the null-hypothesis.

This study consists of two groups of individuals: Patients with SHF with a reduced left ventricular ejection fraction (defined as <40% ejection fraction) and an age-matched healthy control group. SHF was diagnosed clinically according to the Australian SHF guidelines as having typical symptoms and signs that generally occur on exertion, but can also occur at rest (particularly when recumbent) secondary to an abnormality of cardiac structure or function based on echocardiography [1]. Healthy controls were self-reported with no cardiovascular disease or suffering from any comorbidities (e.g., type 2 diabetes, hypertension) and were recruited from university staff, students, and the general population (Brisbane, Queensland, Australia). Exclusion criteria for the study participants were: The existence of any comorbidities (control subjects only); oral disease (e.g., periodontal disease and gingivitis); autoimmune, infectious, musculoskeletal, or malignant disease; and recent operation or trauma. Due to potential ethical concerns, we did not collect blood samples from the healthy controls. The patients with SHF were recruited from the Mater Adult Hospital and the Royal Brisbane and Women’s Hospital under the recommendations of cardiologists. All the patients were diagnosed with SHF by the treating cardiologist using the current Australian clinical guideline [1]. In Australia, it is not standard clinical practice to measure circulating BNP and NT-ProBNP levels as part of the diagnostic workup for patients with SHF. As per the 2018 Australian heart failure guidelines, measurement of natriuretic peptides is generally limited to patients with suspected heart failure where the diagnosis is unclear following initial clinical workup and an echocardiogram cannot be obtained in a timely fashion.

Participants were asked to refrain from eating and drinking (except for water) two hours prior to saliva collection. Resting whole mouth saliva was collected as described previously [23]. In brief, participants were asked to sit in a comfortable position and to rinse their mouths with water. They were asked to tilt their heads down, pool saliva in their mouth for 1 min, and drool into a 50 mL Falcon tube. Saliva samples were aliquoted into 1.5 mL micro centrifuge tubes and stored at −80 °C until analysis. Saliva samples from SHF patients were collected either at the inpatient or outpatient hospital visits and were collected after they received initial treatment. As a consequence, these patients were considered to be in a compensated state at the time of saliva sample collection.

### 2.2. The Development of the Salivary KLK1 and S100A7 ELISA Assays

Capture and detection antibody pairs: a) KLK1 (cat#DY2337-05) were purchased from R&D System (Minneapolis, MN, USA), and b) S100A7 (cat#MBS2101219) were purchased from MyBioSource (Vancouver, BC, Canada). The capture antibody was diluted in phosphate buffered saline (PBS) at 4.0 µg/mL for KLK1 and 2.5 µg/mL for S100A7 only. Then, 100 µL was used to coat 96-well Nunc-Immuno™ MicroWell^TM^ plates (Sigma-Aldrich, St. Louis, MO, USA), then incubated at room temperature overnight. Bio-Plex ProTM II Wash Station (Bio-Rad, Hercules, CA, USA) was used to wash off excess antibodies with 300 µL/well wash buffer (1x PBS and 0.05% Tween20). The plates were then blocked with 1% bovine serum albumin in PBS (1% BSA/PBS) at a volume of 300 µL/well. Coated plates were sealed using the sealing films and kept at 4 °C until required.

A pooled saliva sample was made up of 10 randomly selected saliva samples from healthy individuals and was filtered using an Amicon Ultra-0.5 mL centrifugal filter (cat#UFC5010, Merck Millipore, Billerica, MA, USA) to create analyte-free saliva matrix for KLK1 and S100A7 assays. The filtration efficiency of the Amicon filtration device has previously been evaluated by Johnsen et al. [24] and has demonstrated that by using a 10K filter, protein/peptides that are smaller than 10 kDa can be sufficiently filtered from the human plasma samples. In our study, we collected the filtrated saliva and diluted accordingly to generate the analyte-free diluent for the immunoassays. We compared the standard curve generated using the analyte-free matrix to the standard curves generated using 1 x PBS and found no significant differences in terms of limit of detection, linearity, and recovery.

Seven points standard curves of KLK1: 3000.0 pg/mL–46.9 pg/mL and nine points standard curve of S001A7: 8000.0 ng/mL–2 ng/mL were generated by serially diluting recombinant KLK1 peptide (cat#DY2337-05, R&D System) in 1% KLK1 free saliva matrix and recombinant S100A7 peptide (cat# ab105577, Abcam, Cambridge, UK) in 5% S100A7 free saliva matrix in 1× PBS, respectively. Saliva samples were diluted 50–1000 folds and 10–100 folds for KLK1 assay and S100A7 assay, respectively using 1× PBS. Samples that returned outside of the assay concentration ranges were subsequently diluted so that these samples were well within the assay performance. After washing away the excess assay reagents with the washing buffer, the plate was blocked with 1% BSA/PBS (300 µL/well) at room temperature for 2 h, and the plates were washed with washing buffer. Standards and diluted samples were loaded onto the capture antibody bound 96-well Nunc-Immuno™ MicroWell^TM^ plate, sealed and incubated at room temperature for 2 h. For KLK1 assay, 100 µL of biotinylated anti-KLK1 antibody diluted in 1% BSA/PBS (1 in 60) was added to each well. For S100A7 assay, a total of 100 µL biotinylated anti-S100A7 antibody at a concentration of 0.3 µg/mL (cat#MBS2101219, MyBioSource) was added to each well. The biotinylated antibodies were incubated for 2 h at room temperature. The excess antibodies were washed as before. Diluted streptavidin-HRP (1:10,000 in 1% BSA/PBS, Cat#21130, ThermoFisher Scientific, Waltham, MA, USA) was added to each well and incubated for 30 min at room temperature in the dark. After washing the excess reagent, 100 µL 3,3′,5,5′-Tetramethylbenzidine (TMB) was used as the substrate and the reaction was stopped after 20 min with 50 µL of 2 M hydrogen chloride. The plate was read at 450 nm with a background correction at 540 nm with a Benchmark Plus Microplate Spectrophotometer System (Bio-Rad, Hercules, CA, USA).

### 2.3. The Development of the CAMP ELISA Assay

A pooled saliva sample made up of 10 randomly selected saliva samples from healthy individuals was filtered with an Amicon Ultra-0.5 mL centrifugal filter unit with Ultracel-10 membrane (cat#UFC5010, Merck Millipore, Billerica, MA, US) to create a CAMP-free saliva matrix. A seven points standard curve (1000.0 ng/mL–7.8 ng/mL) was generated by serially diluting recombinant CAMP peptide (cat# ab140725, Abcam, Cambridge, UK) in 5% CAMP free saliva matrix in 1× PBS. Saliva samples were diluted 10–100 folds with 1× PBS. Samples that were out of the assay range were subsequently diluted so that these samples could be accurately measured. The standard and diluted samples were loaded onto a 96-well Nunc-Immuno™ MicroWell^TM^ plate and incubated at 4 °C overnight. After washing the excess unbound reagents, the plate was blocked with 300 µL/well 1% BSA/PBS at room temperature for 2 h. A total of 100 µL anti-CAMP monoclonal antibody at a concentration of 0.5 µg/mL (clone mAbcam58387, cat#ab58387, Abcam, Cambridge, UK) was added into each well and incubated for 2 h at room temperature. The excess antibody was washed away as previously described. HRP conjugated anti-mouse secondary antibody (100 µL per well, 1:5000 dilution, cat#7076, Cell Signaling Technology, Inc., Danvers, MA, USA) was added to each well and incubated for 1.5 h at room temperature in the dark. After washing away excess antibodies, 100 µL of TMB was added to each well and the reaction was stopped after 20 min by adding 50 µL of 2 N M hydrogen chloride. The plate was read at 450 nm with a background correction at 540 nm with Benchmark Plus Microplate Spectrophotometer System (Bio-Rad, Hercules, CA, USA).

### 2.4. Assay Performance Characteristics for the In-House Developed ELISAs

#### 2.4.1. Recovery

To evaluate the suitability of immunoassays for measuring salivary protein levels, we spiked in three different concentrations (high, medium, and low) of recombinant KLK1, S100A7, and CAMP in three saliva samples, respectively, and measured the spiked pooled saliva samples using the in-house developed immunoassays. Neat pooled saliva samples were measured at the same time. The percentage recovery of the three spiked saliva samples was calculated in reference to respective neat pooled saliva samples in a single ELISA, using the following equation [25]:

Percentage Recovery (%) = [(protein concentration in spiked saliva – protein concentration in neat saliva) / (amount of spiked recombinant protein)] × 100.(1)

#### 2.4.2. Limit of Quantification

To determine the limit of quantification (LOQ) of the in-house developed ELISAs, blanks (sample diluent used in each assay) were run in duplicate in one immunoassay run [26]. The LOQ for the salivary ELISAs were read from a sigmoidal four parameters logistic regression based on LOQ signal counts derived from the equation [25]:

LOQ signal count = (average of blank signal count) + 10 × (standard deviation of blank signal).(2)

#### 2.4.3. Intra and Inter-Assay Coefficient of Variation

To determine intra and inter-assay coefficient of variations (CVs), duplicate saliva samples were run in each in-house developed immunoassay. The intra-assay variations were determined by measuring 24 individual saliva samples in triplicate and by calculating the variations based on the measurement. The inter-assay variations were determined by measuring three samples in ten separate assays and by calculating the variations based on the measurements. Intra and inter-assay variations were expressed by intra or inter-assay percent (%CV). The %CV was calculated using the following equation:

%CV=(Standard Deviation×100)÷Mean.

### 2.5. Statistical Analysis

All statistical analyses were performed using GraphPad Prism 8 (GraphPad Software Inc., La Jolla, CA, USA) and R (R Development Core Team. Vienna, Austria). GraphPad was used to generate standard curves for KLK1, S100A7, and CAMP by plotting the raw absorbance values on the *Y*-axis with relevant analyte concentrations [18]. The analyte concentrations were deduced from the standard curve using the sigmoidal four parameters logistic regression equation. For clinical characteristics (continuous variables) of the patients with SHF, the Shapiro–Wilk normality test was performed to test for normal distribution. Logarithmic transformation was performed to normalize the data. The Kruskal–Wallis test and Dunn’s multiple comparisons tests were performed on unpaired data with non-normal distribution to compare values between multiple groups. The diagnostic performance of the three proteins was evaluated by ROC curve analysis.

A multi-model machine learning algorithm was used to generate a prediction model that combined the salivary biomarker measurements with age and gender to improve predictions. The algorithm automatically created and evaluated multiple models with multiple configurations (decision tree, random forests, logistic regressions, and deepnets) by using Bayesian parameter optimization [27], and was split into two parts. The first, “parameter search”, used a single holdout set to iteratively find promising sets of parameters. The second, “validation”, iteratively performed Monte Carlo cross-validation [28] on parameter sets of interest. This split was set to a random 80% training and 20% test dataset. For this second phase, the algorithm iteratively does new train/test splits for the top half of algorithms remaining. Thus, the best models will typically have more than one evaluation associated with them.

Evaluation of the algorithms was sorted by the ROC area under the curve (AUC or diagnostic accuracy) to find the best algorithms. The best performing algorithms were selected to be combined into a single algorithm for the best performance and stability. A total of 64 models (49 random decision forests and 15 logistic regressions) were combined into the final algorithm. The performance of the selected algorithm was validated with 100-fold bootstrap validation method by applying the model generated with a random selection of 70% of the total data set and applying it to a different set of data also generated by random selection of 70% of the total data set.

#### Biological Relevance of the Salivary Protein Panel to Systolic Heart Failure

Data from a previous study [16] (available on ProteomeXchange Consortium via the PRIDE [29] partner repository with the dataset identifier PXD007134), was analysed using gene ontology (GO) enrichment through the STRING v11 application [30] and using pathway enrichment through the Cytoscape (v3.7.0) ClueGO (v2.5.4) app, with KEGG, Reactome, and WikiPathways databases accessed. Only proteins with a significantly differential abundance in the saliva samples from healthy controls and SHF patients were included in the enrichment approaches.

## 3. Results

### 3.1. Participants

In total, 100 patients with SHF and 88 disease-free healthy individuals were recruited into this study. The clinical characteristics of the participants are listed in Table 1.

### 3.2. Assays Performance

The standard curves (average of six independent assays) for each protein is provided in Supplementary Figure S1. The analytical performances of the ELISA assays are summarised in Table 2.

### 3.3. Salivary Protein Measurements

**Figure 1:**
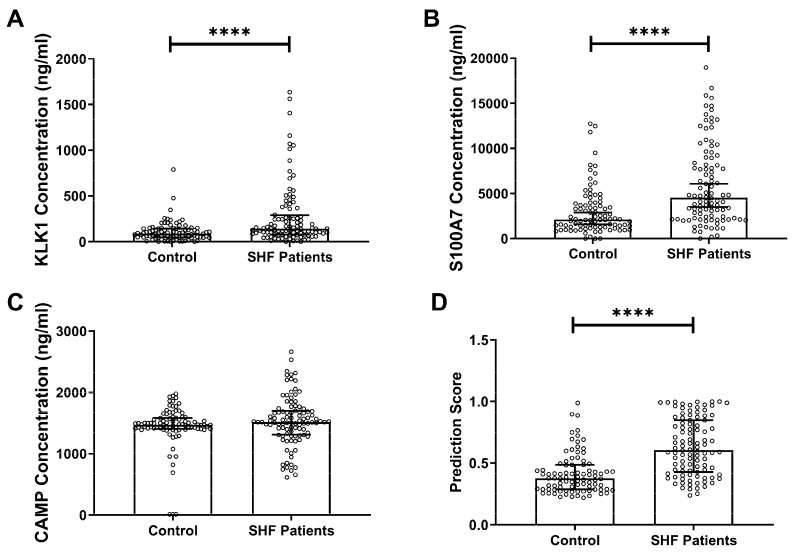
Individual concentrations of (**A**) KLK1, (**B**) S100A7, (**C**) CAMP in saliva samples collected from controls and systolic heart failure (SHF) patients, and (**D**) their combination, plotted on a scatter plot with box and error bars showing the median and 25–75th percentile. The Kruskal–Wallis test and Dunn’s multiple comparisons test were performed on unpaired data with non-normal distribution to compare values between multiple groups. Significant differences in the protein concentrations between patients with SHF and healthy controls are denoted by **** (*p* < 0.0001).

A total of 100 SHF patients’ and 88 healthy controls’ saliva samples were assayed and analysed by immunoassay (Figure 1).

**Figure 2:**
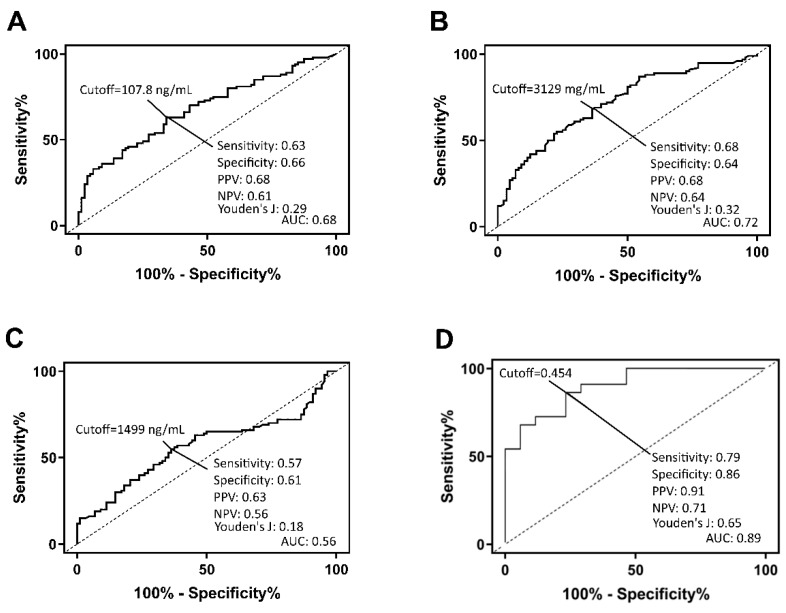
Receiver operating curve (ROC) generated using (**A**) KLK1, (**B**) S100A7, (**C**) CAMP and (**D**) multi-mode machine learning algorithm.

The salivary concentrations of S100A7 in healthy controls ranged from <12.2 ng/mL to 12749.0 ng/mL with a median of 2118.0 ng/mL (IQR, 1280.0–3973.0 ng/mL). The salivary S100A7 levels in patients with SHF ranged from <12.2 ng/mL to 18972.0 ng/mL with a median of 4535.0 ng/mL (IQR, 2282.0–8807.0 ng/mL). The salivary concentrations of CAMP in healthy controls ranged from 10.0 ng/mL to 1978.0 ng/mL with a median of 1471.0 ng/mL (IQR, 1407.0–1580.0 ng/mL). The salivary CAMP levels in patients with SHF ranged from 615.0 ng/mL to 2664.0 ng/mL with a median of 1515.0 ng/mL (IQR, 1311.0–1700.0 ng/mL). The salivary concentrations of KLK1 in healthy controls ranged from <8.0 ng/mL to 789.5 ng/mL with a median of 86.8 ng/mL (IQR: 48.1–143.0 ng/mL). The salivary KLK1 levels in patients with SHF ranged from <8.0 ng/mL to 1636.0 ng/mL with a median of 141.5 ng/mL (IQR: 76.8–289.2 ng/mL). Significant differences in the concentrations of salivary S100A7 (*p* < 0.0001) and KLK1 (*p* < 0.0001) were observed between healthy controls and patients with SHF. We assessed the diagnostic accuracy of each individual marker by ROC curve analysis (Figure 2A–C).

Using the multivariate ROC model [31], we combined the salivary concentrations of KLK1, S100A7, and CAMP into a multi-protein panel. The three-protein panel was able to discriminate a non-disease healthy control group from patients with SHF (*p* < 0.0001, Figure 1D), demonstrating the potential diagnostic utility.

### 3.4. Receiver Operator Characteristic Curve for the Multi-Protein Panel Generated with Multi-Model Machine Learning Algorithm

By combining the gender and age information of the participants using the multi-mode approach, we were able to generate a single algorithm which improved diagnostic accuracy (Figure 2D). The ROC AUC was 0.89, with sensitivity, specificity, positive predictive value, negative predictive value, and overall accuracy at the optimum cut-off point (maximum sensitivity + specificity) being 79.2%, 85.7%, 90.5%, 70.6%, and 81.6%, respectively. The diagnostic performance of the multi-marker protein panel was further refined using the bootstrap validation method. The average sensitivity, specificity, positive predictive value, negative predictive value, and accuracy at the optimal cut-off point (where sensitivity and specificity are closest) were 73.6%, 78.8%, 76.7%, 75.5%, and 76.1%, respectively.

### 3.5. Biological Relevance of the Protein Panel Derived from Saliva Sampling to Systolic Heart Failure

When revisiting the published data (available on ProteomeXchange Consortium via the PRIDE [29] partner repository with the dataset identifier PXD007134) from our previous study [16], we performed a gene ontology (GO) enrichment analysis of the subset of proteins with significantly differential abundance between healthy controls and SHF patients. The GO enrichment revealed significantly overrepresented (false discovery rate (FDR) *p* < 4.5 × 10^−16^) biological processes associated with: Inflammation and immune response (e.g., neutrophil degranulation, FDR *p* = 4.19 × 10^−53^), endopeptidase activity, and epidermal cell differentiation (Supplementary Table S1). This is further supported by pathway enrichment analysis of the same dataset, which revealed significant overrepresentation (FDR *p* < 0.05) of pathways and terms associated with cell adhesion, complement activation, coagulation and the circulation system, and an overall increased inflammatory state (Supplementary Figure S2).

## 4. Discussion

In the present study, using in-house developed immunoassays, we demonstrated the potential clinical utility of a salivary protein panel to detect SHF. With the use of immunoassay, sample preparation and analysis time were dramatically reduced (>24 h sample preparation time; 70 min analysis time per single sample via SWATH-MS vs. 6 h per 40 samples via immunoassay). More importantly, clinical laboratory infrastructure currently uses a wide array of immunoassay platforms for existing clinical purposes, therefore we already have the required resources for these assays as opposed to SWATH-MS. This could facilitate cost-effective, large-scale population screening for SHF. The results from the immunoassays validated our previous findings generated by using SWATH-MS and Western blot analyses [16]. In the current study, a new cohort of SHF patients was assessed and the concentrations of KLK1 and S100A7 in saliva were measured and found to be significantly higher than controls. Although CAMP concentration showed no statistically significant difference between patients and controls, it was previously reported in a higher concentration in controls [16]. Therefore, all three proteins were used in the subsequent logistic regression analysis. We used a multi-mode machine learning algorithm to investigate the diagnostic performance when combing the three proteins in a panel, demonstrating high diagnostic accuracy. Artificial intelligence and machine learning are becoming more mainstream in the field of medical diagnostics and could allow for rapid and accurate interpretation of multiple biomarker measurements.

Enrichment of pathways and gene ontologies within a previously published SHF study dataset revealed significant overrepresentation of enriched biological processes and key pathways that are of relevance to SHF. These overrepresented functions and pathways may reflect the underlying change in biochemistry of the SHF patient saliva proteome, wherein there is a significant enrichment of proteins associated with leukocyte activation, interleukin signalling, infection, and degranulation of neutrophils. Such a response may arise from host processes (cell breakdown/inflammation) and/or external influences, such as infection (viral or bacterial). Overall, these terms may indicate systemic inflammation and an underlying immune challenge that are both enriched in SHF patients. These data support the presence of CAMP and S100A7 as potential biological indicators of the systemic immune response relating to SHF. Furthermore, the significant enrichment of terms associated with both complement and coagulation cascades, platelet degranulation, and endopeptidase regulation may reflect the challenges on the cardiovascular system due to SHF. The enrichment of proteins associated with the vasculature in SHF patients reflects the characteristics of the clinical pathology of this cohort. Moreover, the significant enrichment of endopeptidase regulation supports the presence of KLK1 as a potential biological indicator of SHF.

KLK1 is a serine endopeptidase and part of the family of tissue kallikreins, which are reportedly associated with the presence of coronary artery disease [32]. S100A7, another antimicrobial protein, has been reported to be significantly elevated in patients with psoriasis and atherosclerosis [33]. CAMP is the mature form of the antimicrobial peptide LL-37, of the innate immune system. It is associated with neutrophil degranulation known to be expressed in epithelial tissues [34], and is involved in atherosclerosis where it is reportedly produced in atherosclerotic lesions [35]. Collectively, the three proteins described in the present study have relevant links to the underlying biology present in SHF and have strong links to the pathophysiology of SHF.

This is a preliminary study and needs further validation. We did not collect blood samples from the same patients and healthy controls as the scope of the research was to investigate the feasibility of using saliva as an alternative diagnostic medium compared to the traditional sampling methods. As such, we did not have the matching blood samples from the same patients and controls. Furthermore, due to ethical justifications, we did not collect blood samples from healthy controls. In Australia, it is not standard clinical practice to measure NT-proBNP and BNP as part of heart failure diagnosis and management. All of the patients were diagnosed with heart failure on clinical grounds. Thus, we were unable to compare the diagnostic utility of the salivary biomarker panel with blood-based natriuretic peptide levels. Another limitation to the study was that we did not perform echocardiography on the healthy control group to exclude structural heart disease. Also, we did not have all the imaging and pharmacological data for the SHF patients as well as information pertaining to systemic organ involvement (lung and kidney). Due to the limitation of the information we were able to collect from both patients and healthy controls, we can only include the current parameters in the multi-model panel. CAMP immunoassay developed in-house is not a sandwich ELISA. This is because of the limitations in selecting an antibody pair. The sensitivity and specificity of direct coating ELISA is known to be inferior to sandwiched ELISA [36]. Sandwiched ELISAs could be developed in the future for CAMP with the use of invitro generated antibodies. The current format of the assays requires three separate assays to be performed when quantifying proteins of interest in saliva samples. A multiplex assay combining the measurement of all three proteins could reduce the assay time and sample consumption. This would enable the protein panel to be easily integrated into a primary healthcare setting to reach out to high-risk communities in regional and rural areas. In future validation studies, a multiplex assay should be developed and tested to enable higher throughput measurement of the proteins in saliva samples.

This is the first step in demonstrating the clinical sensitivity and specificity of the panel in a known SHF vs. control cohort. We previously partnered with SCReening Evaluation of the Evolution of New Heart Failure (SCREEN-HF) longitudinal study [37] to include individuals who are at a higher risk of developing heart failure (HF). The inclusion criteria of the SCREEN-HF study includes having at least one HF risk factor (i.e., history of stroke, coronary artery diseases, valvular heart disease, atrial fibrillation, hypertension or diabetes treated for at least two years), or chronic renal impairment (estimated glomerular filtration rate (eGFR) < 60 mL/min/1.73 m^2^). We will measure the three protein concentrations in the saliva samples collected from this cohort in the future to further validate the clinical utility of our multi-marker panel.

## 5. Conclusions

In conclusion, we developed in-house immunoassays to measure the concentrations of KLK1, CAMP, and S100A7 in saliva samples. The assay performances were within the acceptance range. We evaluated the diagnostic power of this three-protein panel by using samples collected from SHF patients and healthy controls. A statistically significant difference of the prediction score was found between the two groups, suggesting that the three-marker panel has the potential to be used as an early diagnostic tool for HF.

## Figures and Tables

**Table 1:** Summary of relevant clinical characteristics for healthy controls and patients with systolic heart failure.

Parameter	Healthy Controls	Systolic Heart Failure Patients
	N = 88	N = 100
Average age (range)	56 (41–92)	67 (29–97)
Gender (%M)	44%	67%
Body mass index (kg/m^2^)	24	33
Previous acute coronary syndrome	N/A	31 (31%)
Hypertension	N/A	38 (38%)
Type 2 diabetes	N/A	36 (36%)
Chronic obstructive pulmonary disease	N/A	16 (16%)

N/A: not available.

**Table 2:** Performance characteristics of the in-house developed enzyme-linked immunosorbent assays (ELISAs).

Performance characteristics		**KLK1**	**S100A7**	**CAMP**
Working range		46.9–3000 pg/mL	2–8000 ng/mL	7.8–1000 ng/mL
Typical sample dilution factor		100	20	20
Goodness of fit (typical R squared)		0.99	0.99	0.99
Spiked-in recovery (spiked in concentration)	High	105.7% (2000 pg/mL)	104.3% (3000 ng/mL)	123.5% (800 ng/mL)
	Median	94.6% (400 pg/mL)	95.1% (600 ng/mL)	84.8% (150 ng/mL)
	Low	90.6% (80 pg/mL)	102.2% (120 ng/mL)	118.7% (30 ng/mL)
Pool saliva sample dilution linearity (percentage recovery)	A	50× dilution: 1609 pg/mL	10× dilution: 994 ng/mL	10× dilution: 426 ng/mL
	B	100× dilution: 801 pg/mL (100%)	20× dilution: 578 ng/mL (116%)	20× dilution: 203 ng/mL (95%)
	C	200× dilution: 366 pg/mL (91%)	50× dilution: 192 ng/mL (96%)	50× dilution: 97 ng/mL (114%)
	D	1000× dilution: 72 pg/mL (89%)	100× dilution: 117 ng/mL (117%)	100× dilution: 39 ng/mL (89%)
Limit of quantification		80.1 pg/mL	12.2 pg/mL	10.0 ng/mL
Inter-assay CV		2.2 ± 1.2%	8.0 ± 1.5%	8.5 ± 2.7%
Intra-assay CV		2.0 ± 0.7%	2.6 ± 0.2%	2.4 ± 0.2%

## Supplementary Materials

The following are available online at https://www.mdpi.com/2218-273X/9/12/766/s1, Figure S1: Typical standard curves of S100A7, KLK1 and CAMP ELISAs. Figure S2: Pathway enrichment analysis, Table S1: Gene Ontology enrichment analysis of biological processes (STRING v11).
