# Supplementary material for: Salivary Protein Panel to Diagnose Systolic Heart Failure
Source: Biomolecules. 2019 Nov 22;9(12):766. doi: 10.3390/biom9120766 (PMC6995570; doi:10.3390/biom9120766)
Supplement: Supplementary file 1 [file biomolecules-09-00766-s001.pdf]

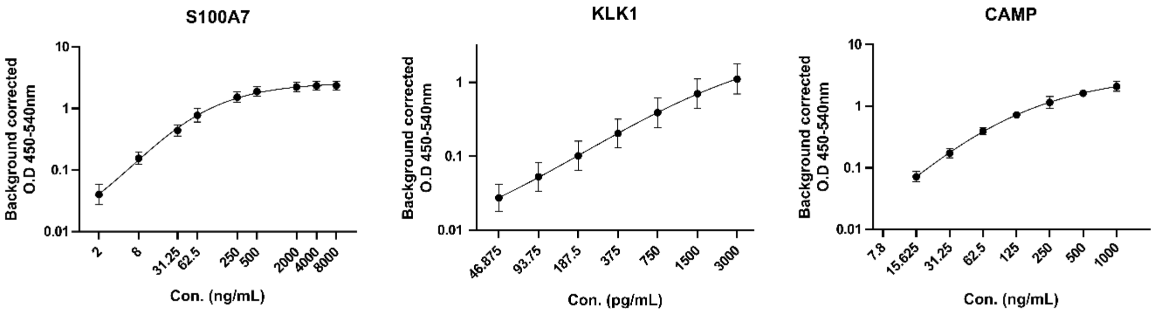

Figure S1: Typical standard curves of S100A7, KLK1 and CAMP ELISAs. The standard curves of each assay was averaged using 6 independent assays.

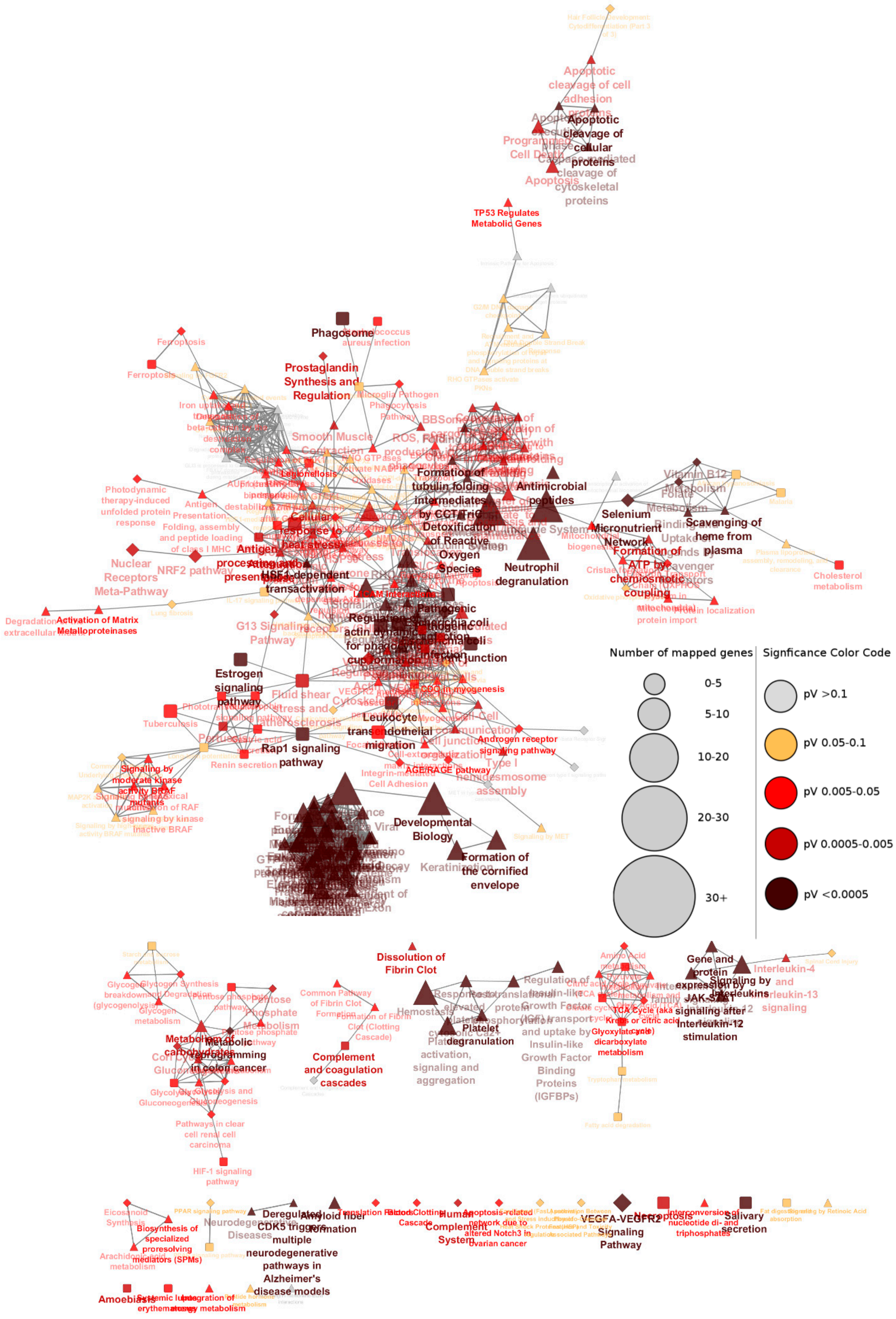

Figure S2: Combining KEGG (round rectangle), Reactome (triangle), and WikiPathways (diamond) through ClueGO (v2.5.4) App in Cytoscape (v3.7.0) revealed a strong and significant overrepresentation (FDR corrected p value < 0.05) of multiple pathways, including Apoptotic

cleavage of cellular proteins, Neutrophil degranulation, Tight junction, Infection, Developmental biology, Interleukin signaling, Platelet degranulation, Complement & coagulation cascades. Collectively the proteome indicates an underlying inflammatory state with strong links to the circulation and cell adhesion.

Table S1: Gene Ontology enrichment analysis of biological processes (STRING v11) showing the top 25 significantly overrepresented terms ($p < 4.5 \times 10^{-16}$). The data show a significant enrichment of proteins involved in biological processes concerning the immune system response, epidermal cell differentiation, and endopeptidase activity.

**#term ID**	**term description**	**observed gene count**	**false discovery rate**
GO:0043299	leukocyte degranulation	83	4.19E-53
GO:0043312	neutrophil degranulation	82	4.19E-53
GO:0045055	regulated exocytosis	93	4.19E-53
GO:0002366	leukocyte activation involved in immune response	86	1.06E-50
GO:0002443	leukocyte mediated immunity	86	5.35E-50
GO:0002274	myeloid leukocyte activation	83	5.95E-50
GO:0002252	immune effector process	95	4.93E-46
GO:0032940	secretion by cell	94	5.63E-44
GO:0045321	leukocyte activation	91	1.20E-43
GO:0046903	secretion	97	6.85E-43
GO:0016192	vesicle-mediated transport	116	4.39E-41
GO:0001775	cell activation	93	5.85E-41
GO:0006955	immune response	108	3.09E-38
GO:0006810	transport	166	4.07E-34
GO:0002376	immune system process	122	6.84E-32
GO:0051179	localization	176	3.61E-27
GO:0070268	cornification	28	3.96E-21
GO:0050896	response to stimulus	205	2.65E-19
GO:0030216	keratinocyte differentiation	34	1.34E-17
GO:0030855	epithelial cell differentiation	49	5.55E-17
GO:0010951	negative regulation of endopeptidase activity	32	5.88E-17
GO:0031424	keratinization	31	7.24E-17
GO:0009913	epidermal cell differentiation	35	7.37E-17

GO:0052548	regulation of endopeptidase activity	38	3.64E-16
GO:0052547	regulation of peptidase activity	39	4.42E-16
